# Formulating RALA/Au nanocomplexes to enhance nanoparticle internalisation efficiency, sensitising prostate tumour models to radiation treatment

**DOI:** 10.1186/s12951-021-01019-8

**Published:** 2021-09-19

**Authors:** Lindsey A. Bennie, Jie Feng, Christopher Emmerson, Wendy B. Hyland, Kyle B. Matchett, Helen O. McCarthy, Jonathan A. Coulter

**Affiliations:** 1 School of Pharmacy, Queen’s University Belfast, Belfast, BT9 7BL Northern Ireland UK; 2 Western Health & Social Care Trust, North West Cancer Centre, Altnagelvin Hospital, Derry/Londonderry, BT47 6SB Northern Ireland UK; 3 Northern Ireland Centre for Stratified Medicine, C-TRIC, Altnagelvin Hospital Campus, Derry/Londonderry, BT47 6SB Northern Ireland UK; 4 School of Chemical Sciences, Dublin City University, Dublin 9, Ireland

**Keywords:** Gold nanoparticles, RALA, Radiosensitisation, Prostate cancer, Nanomedicine

## Abstract

**Background:**

Gold nanoparticles (AuNP) are effective radiosensitisers, however, successful clinical translation has been impeded by short systemic circulation times and poor internalisation efficiency. This work examines the potential of RALA, a short amphipathic peptide, to enhance the uptake efficiency of negatively charged AuNPs in tumour cells, detailing the subsequent impact of AuNP internalisation on tumour cell radiation sensitivity.

**Results:**

RALA/Au nanoparticles were formed by optimising the ratio of RALA to citrate capped AuNPs, with assembly occurring through electrostatic interactions. Physical nanoparticle characteristics were determined by UV–vis spectroscopy and dynamic light scattering. Nano-complexes successfully formed at w:w ratios > 20:1 (20 µg RALA:1 µg AuNP) yielding positively charged nanoparticles, sized < 110 nm with PDI values < 0.52. ICP-MS demonstrated that RALA enhanced AuNP internalisation by more than threefold in both PC-3 and DU145 prostate cancer cell models, without causing significant toxicity. Importantly, all RALA-AuNP formulations significantly increased prostate cancer cell radiosensitivity. This effect was greatest using the 25:1 RALA-AuNP formulation, producing a dose enhancement effect (DEF) of 1.54 in PC3 cells. Using clinical radiation energies (6 MV) RALA-AuNP also significantly augmented radiation sensitivity. Mechanistic studies support RALA-AuNP nuclear accumulation resulting in increased DNA damage yields.

**Conclusions:**

This is the first study to demonstrate meaningful radiosensitisation using low microgram AuNP treatment concentrations. This effect was achieved using RALA, providing functional evidence to support our previous imaging study indicating RALA-AuNP nuclear accumulation.

**Graphic abstract:**

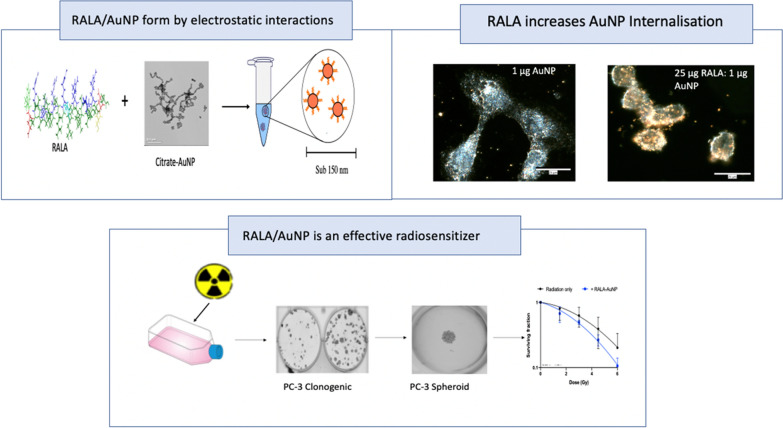

**Supplementary Information:**

The online version contains supplementary material available at 10.1186/s12951-021-01019-8.

## Background

Radiotherapy (RT) is a standard-of-care treatment option for a number of localised cancers including prostate cancer (PCa), offering the best chance for curative intervention [1, 2]. However, 20–40% of PCa patients experience treatment failure leading to disease progression [3, 4]. RT can also contribute to significant off-target damage to neighbouring healthy tissue including the bladder and bowel, impacting patient quality-of-life [5]. As such, efficient radiosensitisers could help improve treatment outcomes, while concurrently limiting off-target radiation damage.

High-Z nanomaterials such as gadolinium (Z = 64), and gold (Z = 79) are proven radiosensitisers, but commercial development leading to widespread use in clinical practice has been hampered by issues that include short systemic circulation, endosomal entrapment and poor target cell uptake [6]. Furthermore, many of the existing pre-clinical studies use nanoparticle concentrations which represent clinically unfeasible doses, both in terms of cost but more importantly posing an elevated risk of systemic toxicity. For example, early in vivo studies demonstrated significant AuNP tumour accumulation and radiosensitisation using 1.9 nm AuNPs using a mammary tumour model. Eighty six percent of mice treated with a single i.v. dose of AuNPs combined with a large 30 Gy dose fraction survived 1-year post treatment compared to only 20% of mice treated with RT alone. However, the clinical relevance of this result is confounded by the high nanoparticle concentration (2.7 g Au/kg) required to produce such a response [7].

One strategy to overcome this limitation is to enhance the efficiency with which AuNPs are internalised using cell penetrating peptides (CPP). CPP are short sequences of amino-acids that facilitate trans-membrane transport through a variety of endocytic pathways, without the requirement of specific targeting receptors [8]. Moreover, the primary and tertiary structure of the CPP can be manipulated to facilitate intra-cellular delivery of various therapeutic cargo [9]. The most promising CPPs possess cationic, basic, amphipathic and fusogenic properties. RALA is an example of such a synthetic CPP, inspired by bio-derived features of naturally occurring CPPs. RALA (sequence—WEARLARALARALARHLARALARALRACEA) is a cationic amphipathic peptide utilised for trans-membrane delivery of a diverse range of anionic cargo [10, 11]. Incorporating arginine repeats confer an overall cationic charge, enabling plasma membrane interaction and internalisation. Furthermore, hydrophilic arginine (R) residues are spatially segregated within its alpha-helical structure, residing on one face of the peptide, with hydrophobic leucine (L) residues located on the other. This confirmation was purposely designed to enhance cell membrane interactions. McCarthy et al., (2014) demonstrated the pH responsive nature of RALA, increasing alpha helicity under acidic conditions, promoting fusogenicity of the peptide/cargo complexes. The pH dependency of RALA occurs as a consequence of glutamate and histidine protonation. At neutral pH COOH groups in the amino acid side chain destabilise the alpha-helical formation, however, in low pH conditions, protonation occurs, increasing hydrophobicity resulting in neutralisation and the formation of an alpha-helix. Increased fusogenicity aids peptide insertion into the endosomal membrane with subsequent cargo release [12]. As such, RALA is a proven delivery platform with the major advantage of conferring minimal direct toxicity [13, 14]. Therefore, it is hypothesised that RALA-AuNP complexes should result an increased AuNP internalisation efficiency, leading to significant radiation dose enhancement in PCa tumour models.

## Results

**Fig. 1:**
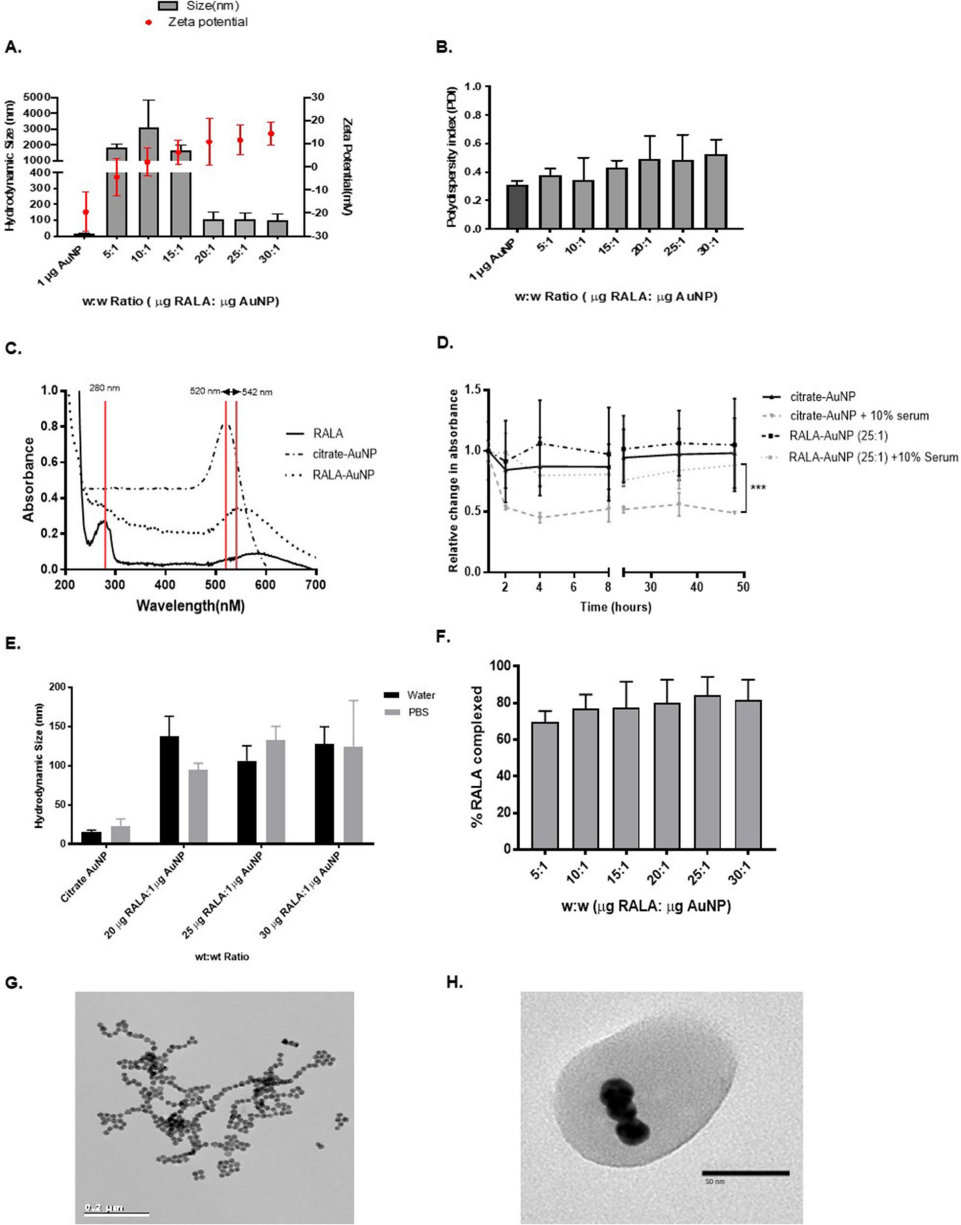
Characterisation of RALA-AuNP complexes. Nanoparticle complexes were prepared at a variety of different w:w ratios from 5 µg RALA: 1 µg AuNP to 30 µg RALA:1 µg AuNP. **A** Hydrodynamic particle size and zeta potential were measured using a Malvern Zetasizer Nano-Zs instrument and DLS software. Mean RALA-AuNP (25:1) hydrodynamic size was 108 nm (± 0.48 nm). **B** Polydispersity index (PDI) was obtained during size measurements. **C** UV-spectrometry used to confirm complexation via alterations in the peak surface plasmon resonance spectra for RALA-AuNP and constituent components. **D** UV–vis assessment of citrate-AuNP and RALA-AuNP stability in serum containing medium. **E** DLS assessment of citrate-AuNP and RALA-AuNP formulation stability in physiological salt conditions. **F** Percentage of complexed RALA to citrate-AuNP (encapsulation efficiency) across a variety of w:w ratios between from 5 µg RALA:1 µg AuNP to 30 µg RALA: µg AuNP. **G** Highly homogenous citrate-AuNPs with core diameter of 15.49 nm (± 2.6 nm). H. TEM image of RALA-AuNP complex at a 25 µg RALA:1 µg AuNP ratio

RALA effectively complexes with negatively charged citrate-AuNPs (− 27 mV ± 11.3) forming sub-110 nm particles at w:w ratios of 20 µg RALA:1 µg AuNP or above (Fig. 1A). Zeta potential measurements of RALA-AuNP increase in line with elevated concentrations of RALA, yielding a mean overall charge of + 18.6 mV for formulations in excess of 20 µg RALA (Additional file 1: Table S1). A positive surface charge promotes electrostatic repulsion of the condensed nanoparticles, preventing agglomeration, an adverse effect observed at w:w ratios of 15:1 or below. PDI values remained constant and below 0.5, indicative of sample homogeneity (Fig. 1B). Changes in the UV–vis spectra were used to confirm complexation. Citrate-AuNPs produce a clear localised surface plasmon resonance (SPR) peak at 521 nm, with RALA alone generating a peak at a wavelength of 279 nm. Following RALA/AuNP complexation a 22 nm SPR shift from 520 to 542 nm occurred. Figure 1C illustrates the various UV–vis spectra for the component parts along with the complexed RALA/AuNP nanoparticles using the 20:1 w:w formulation. Furthermore, a net positive charge confers stability in physiologically relevant serum (Fig. 1D) and salt (Fig. 1E) containing media, limiting agglomeration and providing protection against the formation of a non-specific protein corona. Finally, a BSA assay was used to establish the efficiency of RALA/AuNP complexation. Following synthesis, RALA/AuNP were pelleted and the supernatant collected. A standard curve based on known concentrations of RALA was generated by measuring absorbance at 562 nm. Absorbance values for the supernatant from complexed RALA/AuNP were then used to extrapolate the proportion of uncomplexed RALA, thus providing a measure of complexation efficiency. At w:w ratios of 15 µg RALA:1 µg AuNP and above, more than 80% of the RALA peptide was found to successfully complex with citrate-AuNPs (Fig. 1F). Figures 1G and H represent TEM images of the un-complexed citrate-AuNP metallic nanoparticles (15 nm) along with complexed RALA-AuNPs (25:1 w:w formulation). Formed through non-specific electrostatic self-assembly, negatively charged RALA typically encapsulated between 3 to 5 individual AuNPs (Fig. 1H).

A model radiosensitiser should lack toxicity in the absence of radiation. Direct toxicity conferred by citrate-AuNP and RALA-AuNPs was measured using the fluorescence-based resazurin assay. Surviving fractions (SF) of DU145 and PC-3 tumour cells and PNT2-C2 normal prostate epithelial cells were assessed 6 h post RALA-AuNP treatment (Additional file 1: Figure S1A–C). Dynamic toxicity studies previously published by our group demonstrate biocompatibility of citrate-AuNPs up to 300 µg/ml [15]. A RALA-pEGFP-N1 peptide/DNA complex (hereon termed RALA-GFP) was used as a negative RALA only control, as RALA in the absence of a negatively charged molecule fails to form nanoparticles, promoting naïve state interactions with the cell membrane, triggering direct toxicity. Neither RALA-GFP or RALA-AuNP complexes at w:w ratios up to 30 µg RALA:1 µg AuNP/DNA produced any significant direct toxicity (Additional file 1: Figure S1A–C).

**Fig. 2:**
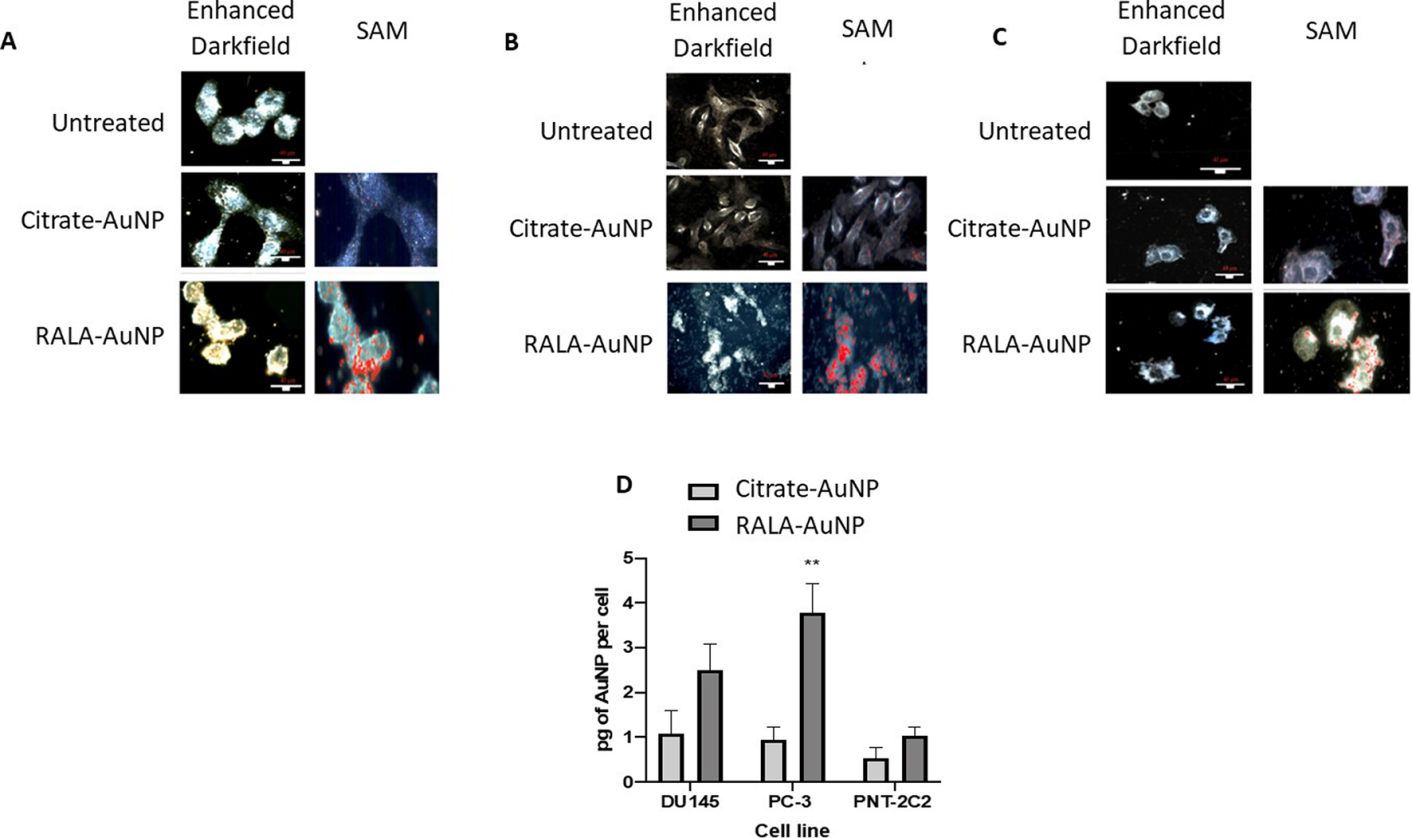
Internalisation of citrate-AuNP and RALA-AuNP complexes in PCa and prostate epithelial cell lines as measured by ICP-MS and Cytoviva. For Cytoviva imaging cells were treated for 6 h with citrate-AuNP and RALA-AuNP at a 25 µg RALA:1 µg AuNP ratio. Representative images include enhanced darkfield and the corresponding hyperspectral images overlaid with the spectral angle map depicting the presence of AuNPs in red. Scale bars are equal to 40 µm. **A** DU145 **B** PC-3 **C** PNT2-C2. **D** For ICP-MS quantification of intracellular Au, cells were treated for 6 h with citrate-AuNP and a w:w ratio of 25 µg RALA:1 µg AuNP. Post treatment cells were trypsinised, dissolved in aqua regia and diluted in molecular grade water. Samples were analysed alongside a series of standards of known concentrations. One-way ANOVA statistical analysis was performed

Citrate-AuNPs and RALA-AuNP complexes were detectable by hyperspectral microscopy in both PC-3 and DU145 cells, with maximum nanoparticle internalisation in PC-3 cells (Fig. 2A and B). Qualitative microcopy studies suggest that RALA significantly enhances AuNP internalisation compared to an equivalent 1 µg of unfunctionalised citrate-AuNP. Interestingly, PNT2-C2 the normal epithelial control cell, internalised less RALA-AuNP complexes, implying preferential tumour specific activity (Fig. 2C). Inductively coupled plasma mass spectrometry (ICP-MS) provides quantitative corroboration of these observations with PC-3 cells exhibiting a significant (p < 0.01) fourfold enhancement in AuNP uptake using RALA. Similar effects were observed in DU145 cells, with both nanoparticle preparations less avidly internalised in PNT2-C2 cells (Fig. 2D). Alexa-Fluor488 dextran nanoparticles were used to establish if the differences between normal and tumour cell internalization could be accounted for by differential endocytic potentials (Additional file 1: Figure S2). Within 2 h post treatment both tumour models demonstrate higher NP internalisation compared to the immortalized normal PNT2-C2 prostate epithelial cells. However, by 6 h post treatment, the duration of RALA-AuNP incubation, nanoparticle uptake had rate reached equivalent levels across all cells tested (Additional file 1: Figure S2B).

**Fig. 3:**
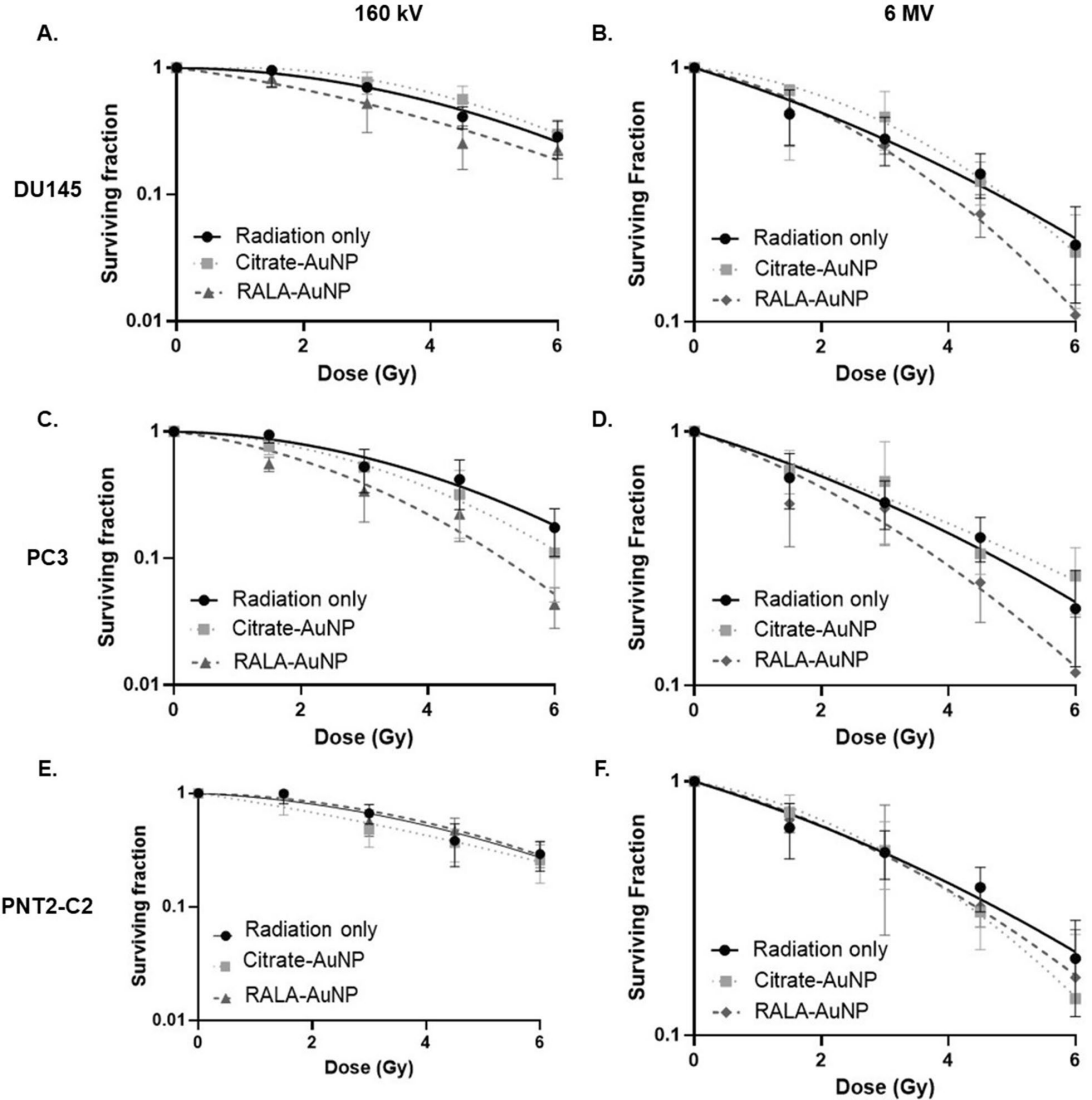
Clonogenic survival assay of prostate cancer and prostate epithelial cells following treatment with RALA-AuNP complexes and radiation. Cells were exposed to 1 µg of citrate-AuNP or RALA-AuNP (25:1 w:w) for 6 h. Excess nanoparticles were then removed and cells irradiated with 0–6 Gy (160 kV—**A**, **C**, **E** or 6 MV—**B**, **D**, **F**) then incubated under normal cell culture conditions for 12 days before scoring. Panels represent cell survival with various gold treatments compared to radiation alone. **A** and **B** DU145; **C** and **D** PC-3; **E** and **F** PNT2-C2. A linear quadratic (LQ) curve fit was applied

To assess the radiosensitising potential of RALA-AuNP experiments were performed using both kV (Fig. 3A, C and E) and MV (Fig. 3B, D and F) photon sources. MV experiments were conducted to address concerns that physical dose enhancement using nano-sized, high-Z elements dominate in the kV range but are diminished at MV energies, an assumption attributed to the limited differential between the mass absorption co-efficient for high atomic number elements and soft tissue (i.e., tumours) at clinical energies. Full details of the clinical radiation experimental setup and dosimetry are provided in Additional file 1: Figure S3A and B.

We compared the sensitising efficacy of a 1 µg/ml treatment concentration for citrate-AuNP against an equivalent AuNP concentration complexed with RALA. Citrate-AuNP yielded a maximum sensitser enhancement ratio (SER) of 1.14 in PC3 cells (Fig. 3C), an effect not replicated in Du145 cells. Similarly, low treatment concentrations of 1 µg/ml citrate-AuNP failed to confer any doe modifying effects using MV X-ray energies. Furthermore, while effective in providing proof-of-concept data for AuNP mediated radiosenstitsation, unfunctionalised citrate-AuNPs lack sufficient stability against serum induced agglomeration to prove effective in a physiological context. Conversely, RALA-AuNP formulations yield significant (p < 0.01) sensitiser enhancement ratios using both kV (SER 1.54) and MV (SER 1.31) radiation sources in PC3 cells (Fig. 3C; Tables 1 and 2). Similar radiosensitising effects, albeit the magnitude of effect was slightly tempered, were observed in DU145 cells (Fig. 3A and B), while the impact in PNT2-C2 cells was limited, correlating with the lower nanoparticle internalisation observed in the PNT2-C2 cells. To confirm these data were the result of RALA-AuNP and not an unexpected effect caused by the RALA peptide, we repeated the kV clonogenic experiments using the RALA-GFP formulation (Additional file 1: Figure S4A–C). Unsurprisingly RALA-GFP failed to significantly augment the effect of radiation treatment, however, equivalence in the survival curves further highlight the biocompatible nature of RALA delivery peptide. Analysis of the linear quadratic fit to extrapolate the PC3 α and β components indicate a marked increase in the α component, indicative of increased direct damage, implicating DNA damage as a primary target for RALA-AuNP radiosensitisation (Table 1). Furthermore, the magnitude of RALA-AuNP radiosensitisation increased with dose (Table 1), the implications of which are important given the shift in clinical practice towards fraction size dose escalation following the successful CHHiP, RTOG and PROFIT trials.

**Table 1:** Sensitiser enhancement ratios (SER) for prostate cancer and prostate epithelial cells treated with citrate-AuNP or RALA-AuNP conjugates

	Mean SER	DEF @ 2 Gy	DEF @ 4 Gy	α	β
**DU145**					
Radiation	–	–	–	0.15 ± 0.05	0.08 ± 0.007
Citrate-AuNP	0.9	0.9	0.85	− 0.06 ± 0.04	0.04 ± 0.01
RALA-AuNP	1.23	1.22	1.49	0.11 ± 0.07	0.03 ± 0.02
**PC-3**					
Radiation	–	–	–	0.08 ± 0.07	0.032 ± 0.02
Citrate-AuNP	1.14	1.14	1.22	0.147 ± 0.05	0.028 ± 0.02
RALA-AuNP	1.54	1.66	2.04	0.35 ± 0.06	0.006 ± 0.02
**PNT2-C2**					
Radiation	–	–	–	0.199 ± 0.04	0.04 ± 0.01
Citrate-AuNP	1.1	1.17	1.2	0.093 ± 0.091	0.028 ± 0.22
RALA-AuNP	0.96	0.97	1.07	− 0.045 ± 0.15	0.0053 ± 0.0039

SER represents a ratio of the area under the curve for control verses treated cells using a survival fraction baseline of 0.1. DEF provides a ratio of survival fractions for control/AuNP treated at a single fixed dose point e.g., 2 Gy and 4 Gy, the survival fractions of which are extrapolated from the LQ survival curve. Similarly, α and β values have been extrapolated from LQ survival curves

**Table 2:** Comparison of sensitiser enhancement ratios (SER) obtained using 160 kV and 6 MV X-ray sources

	160 kVp	6 MV
**DU145**		
+ 1 µg Citrate-AuNP	0.9	1
+ RALA-AuNP	1.23	1.21
**PC-3**		
+ 1 µg Citrate-AuNP	1.15	1.04
+ RALA-AuNP	1.54	1.31
**PNT2-C2**		
+ 1 µg Citrate-AuNP	1.1	1
+ RALA-AuNP	0.96	1.014

**Fig. 4:**
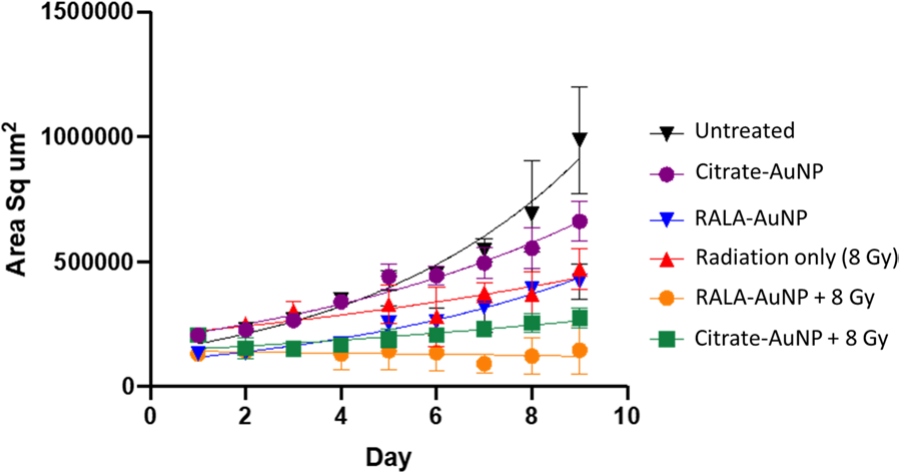
Growth rate of PC-3 spheroids following treatment with RALA-AuNP and Radiation. 1000 PC-3 cells were seeded in a round bottom 96-well plate for 48 h, allowing tumorspheres to form. Tumorspheres were treated with citrate-AuNP or RALA-AuNP complexes at a w:w ratio of 25:1 µg for 6 h, then irradiated with 8 Gy using 160 kV X-rays. Tumoursphere growth rate and doubling time was measured daily using the Celli3Imager. Each treatment group corresponds to 40 spheroids

Three-dimensional tumorspheres provide an animal free means for assessing the potential of experimental therapeutics in a model that possess barriers to free diffusion and naturally occurring oxygen gradients, thereby more closely replicating the tumor microenvironment. PC-3 tumorspheres were treated with RALA-AuNP (25 µg RALA:1 µg AuNP) or citrate-AuNP for 6 h prior to radiation treatment using a single 8 Gy dose. Spheroid growth was measured daily over 8 days (Fig. 4). The nature of these assays mean that media containing excess nanoparticles cannot be completely removed due to the risk of disturbing spheroid growth. As such, developing tumorspheres are exposed to nanoparticle formulations throughout the course of the assay. Despite long-term exposure, RALA-AuNP in the absence of radiation minimally affected the mean doubling time (4.2 days verses 3 days) over untreated controls (Additional file 1: Table S2). Radiation alone (8 Gy) increased spheroid doubling time by 2.5-fold (8.3 days verses 3.3 days) over unirradiated controls. Combined with radiation, citrate-AuNP further extended tumorsphere doubling time by almost 2 days, although not statistically significant compared to radiation alone (Fig. 4 and Additional file 1: Table S2). Validating the 2-dimentional clonogenic assays, RALA-AuNP acted as a potent radiosensitiser, the impact of which was that tumorsphere growth was incalculable with an overall reduction in tumorsphere volume recorded (Fig. 4).

**Fig. 5:**
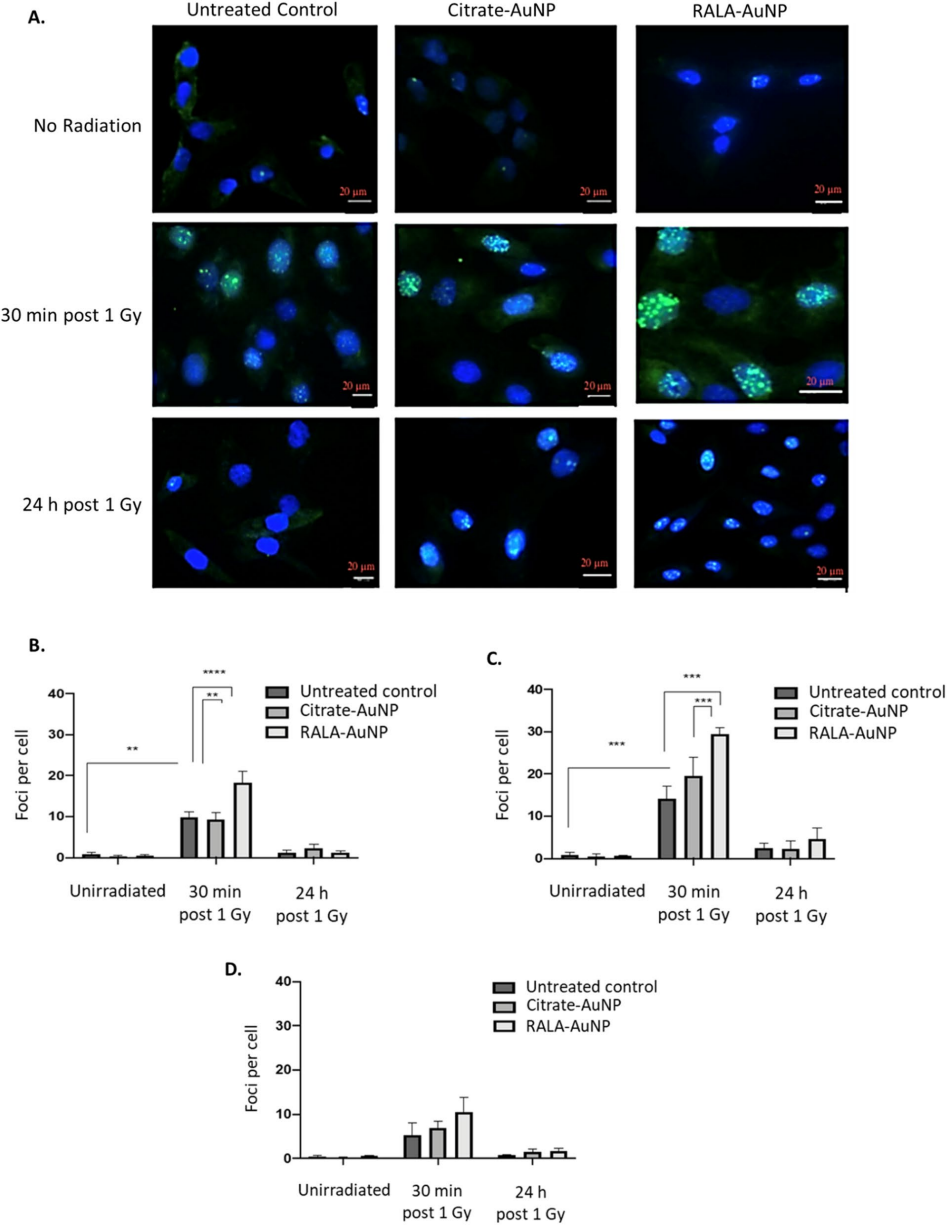
Quantification of DNA double strand breaks following radiation and nanoparticle treatment. Cells seeded in chamber slides were treated with citrate-AuNP (1 µg) or RALA-AuNP (25 µg RALA: 1 µg AuNP) for 6 h. Excess AuNPs were removed, cells washed and then irradiated with 1 Gy. Cells were fixed and stained with anti-53BP1 and DAPI 30 min and 24 h post irradiation. Slides were imaged using a Lecia SP8 confocal microscope using × 40 objective. Z-stacks were taken at 0.5 µm steps with a minimum of 12 sections acquired. **A** Representative images of three independent replicates for PC-3 cells. Quantified 53BP1 foci indicating double strand break lesions in: **B** DU145, **C** PC-3 and **D** PNT2-C2 cells

DNA double strand break damage was assessed using 53BP1 foci formation as a surrogate marker of DNA breaks (Fig. 5A). These experiments help establish if RALA-AuNP radiosensitisation was in part caused by increased DNA damage yields. Citrate-AuNP or RALA-AuNP alone had no effect on DNA damage in any of the cell lines tested (Fig. 5B–D). Unsurprisingly, radiation alone (1 Gy) increased DNA DSB induction in both PC-3 and DU145 cells 30-min post treatment. However, RALA-AuNP significantly augmented DNA DSB lesions over radiation alone in both PC-3 (p-value < 0.0001) and DU145 (p-value < 0.0005) cells. By 24 h post-radiation treatment, in PC-3 cells where maximal radiosensitisation was observed, there remained a higher proportion of unresolved DSB lesions, indicating increased lesion complexity, accounting for the reduced clonogenic survival observed in these cells (Fig. 5A and C). 53BP1 foci data in PNT2-C2 cells support the earlier findings of minimal normal cell uptake with no RALA-AuNP or citrate-AuNP radiosensitisation (Fig. 5D). Importantly, data indicating increased DNA damage and lesion complexity, corroborate our earlier findings which demonstrate RALA mediated nuclear transport of AuNPs, an effect further supported by ICP-MS quantification of total Au content within nuclear and cytoplasmic compartments in RALA-AuNP treated cells (Additional file 1: Figure S5A and B).

## Discussion

Translation of pre-clinical AuNP formulations have been impeded by poor stability and low cell internalisation [16, 17]. Many studies have demonstrated the radiosensitising potential of nanogold, however, in most cases this is at concentrations that lack clinical feasibility, due to concerns of systemic retention, off-target toxicity and cost [7]. This study was designed to address these barriers by using the RALA delivery system to enhance AuNP internalisation and radiation sensitivity.

Citrate-AuNP functionalisation with RALA occurs by straightforward electrostatic self-assembly due to the opposing charge of each component [7]. UV-spectrometry data for RALA-AuNP indicate a visible shift in the UV–vis SPR peak from 520 to 529 nm (Fig. 1). Successful electrostatic complexation and condensation into sub-100 nm particles occurs at w:w ratios > 20:1. Conversely, formulations composed of lower RALA ratios possess a closer to neutral zeta potential, promoting agglomeration and nanoparticle instability.

There are several motivating factors for enhancing tumour specific AuNP uptake. These include reduced nanoparticle treatment concentrations and elevated DNA damage due to enhanced tumour loading. Both factors contribute towards the ultimate goal of widening the therapeutic window between radiotherapy efficacy and treatment associated toxicity, thus improving patient outcome. Quantitative ICP-MS and qualitative imaging techniques both demonstrate that RALA greatly enhances the efficiency of AuNP internalisation over un-complexed citrate-AuNP. Of the formulations investigated, maximal uptake efficiency was observed using the 25:1 w:w ratio. Due to the fact that RALA operates as a non-specific CPP, small levels of Au were detectable in the normal PNT2C2 cells, an effect that was anticipated [18]. Despite this, PNT2-C2 Au levels were significantly lower compared to both tumour cell lines. Similar findings were reported with a 60 nm PEGylated nanoparticle, with the authors reporting greater squamous carcinoma cell internalisation compared to normal keratinocytes [19]. This difference may be in part attributed to differential endocytic potentials between tumour and non-tumour cells. By examining early time points post AuNP treatment, PC-3 and DU145 cells exhibited threefold greater intracellular Au concentrations relative to PNT2-C2 cells, indicating a higher rate of endocytosis in tumour cells. However, over time the differential response was lost, suggesting that in vitro nanoparticle internalisation reaches a point of saturation.

When used in combination with radiation, RALA-AuNP acts as an efficient radiosensitiser, achieving dose enhancement effects using AuNP treatment concentrations at least two orders of magnitude below previously published experimental and computational models of AuNP radiosensitisation [15, 20, 21]. In PC-3 cells, RALA:AuNP (25:1) produced an overall SER of 1.54, representing a highly significant enhancement of radiation sensitivity. Survival curve analysis indicate the effect is dominated by an increase in direct damage, implying that the presence of AuNP directly augments DNA damage. Furthermore, irrespective of radiation energy, the dose enhancement effects of RALA-AuNP were further augmented by dose fraction size, illustrating significant potential in respect to hypofractionated regimes, and aligning closely with recent changes in clinical practice [22, 23].

3D-prostasphere assays mimic some characteristics of a developing tumour including the presence of naturally occurring oxygen and nutrient diffusion gradients, commonly observed with regions of hypoxia/anoxia [24, 25]. Mikahal et al. reported that a 40-fold higher docetaxel concentration was required to achieve a 50% reduction of HELA cell growth using a spheroid assay compared to conventional 2-dimentional monolayer experiments. This differential is likely due to poor drug penetration beyond a few cell layers. To address the issue of drug penetration the authors formulated docetaxel into a polymeric micelle markedly lowering the effective concentration [26]. In the current study, RALA efficiently augments nanoparticle uptake, allowing the use of lower nanoparticle concentrations. At the point of radiation treatment a spheroid contains approximately 50,000 cells, weighing around 5 μg. Using the ICP-MS gold mass data (Fig. 2D), this translates to a gold loading per prostasphere of approximately 0.004%, a likely overestimation as the ICP-MS data was collected from cells grown in monolayers. This level of gold loading is approximately 250-fold lower than the 1% gold loading reported necessary to achieve a doubling of the dose enhancement effects using Monte Carlo simulations [27]. However, we present a doubling of the dose modifying response at 4 Gy. From a physical perspective, AuNP radiosensitisation is directly linked to increased Auger electron release and the photoelectric effect. McMahon et al. developed an in silico radiation transport nanodosimetry model indicating that low energy Auger electrons possess a range of approximately 10 nm [28]. At high AuNP concentrations, saturation and nanoparticle clustering occur, increasing ionisation events deep within the aggregated nanoparticle cluster, rather than within the cytoplasmic/nuclear milieu. This means that secondary electron species will lack sufficient energy to escape the core of the cluster, attenuating the ability of the liberated electrons to induce biological damage. Importantly, the current dataset indicates elevated unresolved DNA damage following RT, supporting our earlier imaging study which reports nuclear accumulation of RALA-AuNP, which highlight the importance of AuNP localisation (Additional file 1: Figure S5B) [15, 29, 30].

While DNA damage undoubtedly makes an important contribution to RALA-AuNP mediated damage, it would be remiss to conclude that this is the sole mechanism responsible for the observed radiosensitisation. Recent studies have identified damage to key organelles such as the mitochondria or lipid peroxidation to the cell membrane, arising from elevated ROS as important contributing factors [31–33]. As such, follow-on studies will focus on the additional contributions of such mechanisms.

Central to this study is the fact that RALA dramatically increases the efficiency of AuNP internalisation. The wider implication of this may ultimately facilitate clinical radiosensitisation using feasible AuNP treatment concentrations. Within the current study we report significant radiosensitisation using RALA-AuNP, crucially at clinical MV radiation energies. Taking into consideration our previous report of RALA-AuNP nuclear accumulation, enhanced DNA damage yields were identified as a key mechanism for the observed increase in radiation sensitivity.

## Materials and methods

### RALA

RALA was produced via solid state synthesis (FMOC chemistry) (Biomatik, UK), supplied as a lyophilised powder and stored at − 20 °C until resuspension. Peptides were resuspended in DNase/RNase free water (Life technologies, UK) and were supplied in acetate salt with a purity > 95%.

### Citrate gold nanoparticle synthesis

Core citrate-AuNPs were synthesised using the widely established Turkevitch/Frens method [34]. 24 h post synthesis particle formation was confirmed using UV–Vis, with physical properties including size, charge and PDI established using dynamic light scattering (DLS).

### Cell culture

PC-3 and DU145 PCa cells were purchased from American Type Culture Collection (ATCC) UK and authenticated via short tandem repeat (STR) profiling. The prostate epithelial cell line PNT2-C2 was kindly supplied by Professor Norman Maitland (University of York). Cells were maintained in RPMI 1640 medium (Gibco,UK), supplemented with 10% fetal bovine serum (FBS) (Gibco, UK). All cells were maintained in monolayers and sub-cultured to 70–90% confluence to ensure exponential growth. Cells were routinely tested to ensure mycoplasma free status.

### Radiation source

kV radiation experiments were performed using a 160 kVp X-ray source (Faxitron CP-160 Arizona USA) with a 0.8 mm Be filter. All doses stated are the absorbed dose in water 33 cm from the radiation source, at a dose rate of 0.77 Gy/min. MV irradiations were performed using a Varian TrueBeam™ linear accelerator (LINAC) in the North West Cancer Centre’s Radiotherapy Facility (Altnagelvin Hospital, Western Health and Social Care Trust). To achieve dosimetric accuracy, an in-house phantom was produced for irradiation purposes and treatment plans generated using Eclipse™ treatment planning system (Additional file 1: Figure S3).

## Methods

### Nanoparticle preparation

RALA-AuNP complexes were prepared at a variety of w:w ratios with the total Au mass maintained at 1 µg. RALA concentrations were increased from 1 to 30 µg, with the final volume adjusted using water to 50 µl. Samples were then incubated at room temperature for 30 min to allow nano-complex self-assembly.

### Nanoparticle physical characterisation and stability

UV–vis spectrometry was used to confirm both citrate-AuNP synthesis and RALA-AuNP complexation. Hydrodynamic particle size was measured via DLS using a Nano-Zs-Zetasizer (Malvern instruments, UK). Poly-dispersity index (PDI), a measurement of sample homogeneity, was also obtained during size measurements. Zeta potential measurements were used to establish nanoparticle surface charge. A BSA protein assay using a RALA standard curve was used to determine the percentage of RALA that complexes with citrate-AuNP. Following centrifugation (12,000 rcf for 30 min) un-complexed RALA within the supernatant was measured by absorbance at 562 nm and compared relative to the total RALA in the self-assembly reaction. Absorbance measurements were acquired using the FLUOstar Omega multi-well plate reader.

### In vitro toxicity assays

1 × 10^4^ cells were seeded and left to adhere overnight in a 96 well plate (Nunc,UK). Cells were treated for 6 h with citrate-AuNP, RALA-AuNP or RALA-GFP (used as a RALA only control), after which 100 µl of complete medium was added. 24 h post nanoparticle treatment, culture media was spiked with 10% resazurin and incubated at 37 °C for 4 h before reading fluorescent conversation as a direct measure of metabolically active cells.

### Inductively coupled plasma mass spectroscopy (ICP-MS)

1 × 10^5^ cells were seeded in 6 well plates (Nunc, UK) and left to adhere overnight at 37 °C in 95%air/5% CO_2_. Cells were then washed using 1 ml of PBS, after which nanoparticle complexes were added. Following a 6 h incubation period, excess non-internalised particles were removed by washing with PBS, cells trypsinised and counted. Samples were dissolved in 1 ml aqua regia, then diluted in water to a total volume of 5 ml. Au quantification was measured using ICP-MS Thermo Scientific iCap Q ICP-MS connected to a CETAC ASX-520 auto-sampler. A linear series of Au only calibration standards were prepared at 0, 1, 10 and 100 ppb made up in 1% HNO_3_/1%HCl (by volume). A quality control standard QC was analysed every tenth sample. Only samples meeting the precise calibration and acceptable QC recoveries were reported.

### Cytoviva enhanced darkfield hyperspectral imaging

Enhanced darkfield microscopy coupled with hyperspectral imaging was performed using the CytoViva microscope (CytoViva Inc,Auburn,Al,USA). Hyperspectral scanning creates a spectral library highlighting unique spectral differences on a pixel-by-pixel basis between untreated control and treated samples. Spectral libraries are applied to darkfield images of treated samples to produce a spectral angle map (SAM) depicting areas of AuNP accumulation. 2 × 10^4^ cells were seeded onto 4-well glass chamber slides (Nunc, UK) and treated with citrate-AuNPs or RALA-AuNP for 6 h. Cells were then fixed using 4% paraformaldehyde before mounting using Vectashield containing DAPI (Sigma, UK).

### 2D clonogenic assays

Survival fractions were determined using the clonogenic assay, as previously described [35]. Briefly, cells were exposed to RALA-AuNP, citrate-AuNP or RALA-GFP for 6 h, excess particles removed, then irradiated using doses between 0 and 6 Gy. Radiation survival fractions were subsequently fitted to the linear quadratic model, from which α/β ratios, sensitiser enhancement ratios (SER) and dose enhancement factors (DEF) were calculated.

### 3D radiosensitisation assays

1 × 10^3^ PC-3 cells were seeded in a round bottomed low attachment 96-well plate, with the complete media volume topped up to 200 µl. Cells were incubated for 48 h to allow tumorsphere formation. Tumourspheres were exposed to citrate-AuNP or RALA-AuNP for 6 h before radiation treatment using a single 8 Gy dose. Tumoursphere growth was measured daily using the Celli3Imager (Screen, Japan). Growth media was replenished every four days by replacing 100 µl (50%) of media with fresh complete medium. Average tumorsphere volume was plotted and exponential growth analysis was carried out using Prism 8.0. Tumoursphere doubling time was calculated from the fitted exponential growth curve.

### DNA double strand break damage using 53BP1

2 × 10^4^ cells were seeded in 4-well glass chamber slides (Nunc,UK) and left to adhere for 48 h. Cells were incubated with citrate-AuNP or RALA-AuNP for 6 h before removal of non-internalised nanoparticles and treatment with a 1 Gy radiation dose. Cells were fixed in 4% formaldehyde (Sigma, UK) after either 30 min or 24 h post radiation treatment. Following permeabilisation (0.1% Tween20 in PBST) and blocking (1% BSA in PBST), samples were incubated for 1 h in primary 53BP1 Ab (1:1000 dilution in 2% BSA) (Abcam, UK). Subsequently, cells were washed in PBS and incubated with Alexafluor 488 secondary mouse antibody for 1 h (1:1000 dilution in 2% BSA—Abcam, UK). Finally, cells were mounted using Vectashield containing DAPI (Invitrogen, UK). Fifty cells were scored per condition per replicate under a Lecia SP8 confocal microscope.

### Statistical analysis

All results presented are the mean of at least three individual experiments ± standard error of the mean (SEM). Data was plotted using Prism 8.0. Depending upon treatment conditions a range of statistical tests were used including one-way and two-way ANOVA, and unpaired two-sample *t*-test. Data were considered significant when p < 0.05.

## Supplementary Information

**Additional file 1: Figure S1.** Toxicity profiles of RALA-AuNP complexes in PCa cell lines. Direct cytotoxicity was determined using the resazurin based Alamar blue assay. **A.** DU145, **B.** PC-3 and **C.** PNT2-C2 cells were treated for 6 h with various w:w ratios of RALA-AuNP up to 30 µg RALA: 1 µg AuNP. Post treatment (24 h) 10% resazurin was added to medium and fluorescence conversion measured. RALA-GFP complexes were used as negative control to assess the direct cytotoxicity of the RALA delivery system. **Figure S2.** Differential endocytosis rates between tumour and non-cancer prostate cell lines. DU145, PC-3 and PNT2-C2 cell lines were treated with Alexa-Fluor488 dextran nanoparticles at a concentration of 5 µM. Samples were collected over a 6 h time course for flow cytometry analysis measuring the percentage of fluorescent positive cells. **A** Comparison of endocytosis rates 10 min post treatment. **B** Comparison of endocytosis rates 6 h post treatment. **Figure S3. A.** Schematic representation of the experimental setup used for cell irradiations with 6 MV photons on a Varian TrueBeam™ LINAC. **B.** MV irradiations were carried out at the North West Cancer Centre using a Varian TrueBeam™ LINAC. For irradiation purposes, to achieve dosimetric accuracy, an in-house phantom was constructed, scanned and planned (Eclipse™ treatment planning system, AcurosXB 13.6.23) for the dose range investigated. Detailing the planned dose distribution in colour wash, the dose prescription point and the dose profile across the plane of the cells. **Figure S4**. Clonogenic survival assay of prostate cancer and prostate epithelial cells following treatment with RALA-GFP complexes and radiation. Cells were exposed to RALA-GFP at a w:w ratio of 20 µg RALA: 1 µg pEGFP-N1 plasmid DNA for 6 h (Panels A-C). Cells were irradiated with 0–6 Gy and left for 12 days before staining with crystal violet and counted. A linear quadratic (LQ) curve fit is applied. **Figure S5.** Validation of nuclear accumulation. **A.** Purified cytoplasmic and nuclear fractions from DU145 cells were collected using differential centrifugation. Nuclear lamins in type V intermediate filaments are highly conserved within the nucleus. Western blot analysis used to confirm purity of nuclear/cytoplasmic isolates. **B.** ICP-MS quantification of total Au content in nuclear and cytoplasmic fractions. **Table S1.** Physiochemical characteristics of AuNP formulations determined by dynamic light scattering. **Table S2.** Doubling time of PC-3 tumorspheres following treatment with citrate-AuNP and RALA-AuNP in the absence and presence of radiotherapy.

## Data Availability

Raw data will be made freely available upon request. Methods contained within or published elsewhere.

## Abbreviations

AuNP: Gold nanoparticles
CPP: Cell penetrating peptide
DLS: Dynamic light scattering
DSB: Double strand break
DEF: Dose enhancement factor
ICPMS: Inductively couples plasma mass spectrometry
PDI: Polydispersity index
PCa: Prostate cancer
RT: Radiotherapy
SER: Sensitiser enhancement ration
SPR: Surface plasmon resonance
