# Supplementary material for: Formulating RALA/Au nanocomplexes to enhance nanoparticle internalisation efficiency, sensitising prostate tumour models to radiation treatment
Source: J Nanobiotechnology. 2021 Sep 19;19:279. doi: 10.1186/s12951-021-01019-8 (PMC8451112; doi:10.1186/s12951-021-01019-8)
Supplement: Supplementary file 1 — Additional file 1: Figure S1. Toxicity profiles of RALA-AuNP complexes in PCa cell lines. Direct cytotoxicity was determined using the resazurin based Alamar blue assay. A. DU145, B. PC-3 and C. PNT2-C2 cells were treated for 6 h with various w:w ratios of RALA-AuNP up to 30 µg RALA: 1 µg AuNP. Post treatment (24 h) 10% resazurin was added to medium and fluorescence conversion measured. RALA-GFP complexes were used as negative control to assess the direct cytotoxicity of the RALA delivery system. Figure S2. Differential endocytosis rates between tumour and non-cancer prostate cell lines. DU145, PC-3 and PNT2-C2 cell lines were treated with Alexa-Fluor488 dextran nanoparticles at a concentration of 5 µM. Samples were collected over a 6 h time course for flow cytometry analysis measuring the percentage of fluorescent positive cells. A Comparison of endocytosis rates 10 min post treatment. B Comparison of endocytosis rates 6 h post treatment. Figure S3. A. Schematic representation of the experimental setup used for cell irradiations with 6 MV photons on a Varian TrueBeam™ LINAC. B. MV irradiations were carried out at the North West Cancer Centre using a Varian TrueBeam™ LINAC. For irradiation purposes, to achieve dosimetric accuracy, an in-house phantom was constructed, scanned and planned (Eclipse™ treatment planning system, AcurosXB 13.6.23) for the dose range investigated. Detailing the planned dose distribution in colour wash, the dose prescription point and the dose profile across the plane of the cells. Figure S4. Clonogenic survival assay of prostate cancer and prostate epithelial cells following treatment with RALA-GFP complexes and radiation. Cells were exposed to RALA-GFP at a w:w ratio of 20 µg RALA: 1 µg pEGFP-N1 plasmid DNA for 6 h (Panels A-C). Cells were irradiated with 0–6 Gy and left for 12 days before staining with crystal violet and counted. A linear quadratic (LQ) curve fit is applied. Figure S5. Validation of nuclear accumulation. A. Purified cytop [file 12951_2021_1019_MOESM1_ESM.docx]

**Additional file 1:**

**Figure S1**

**
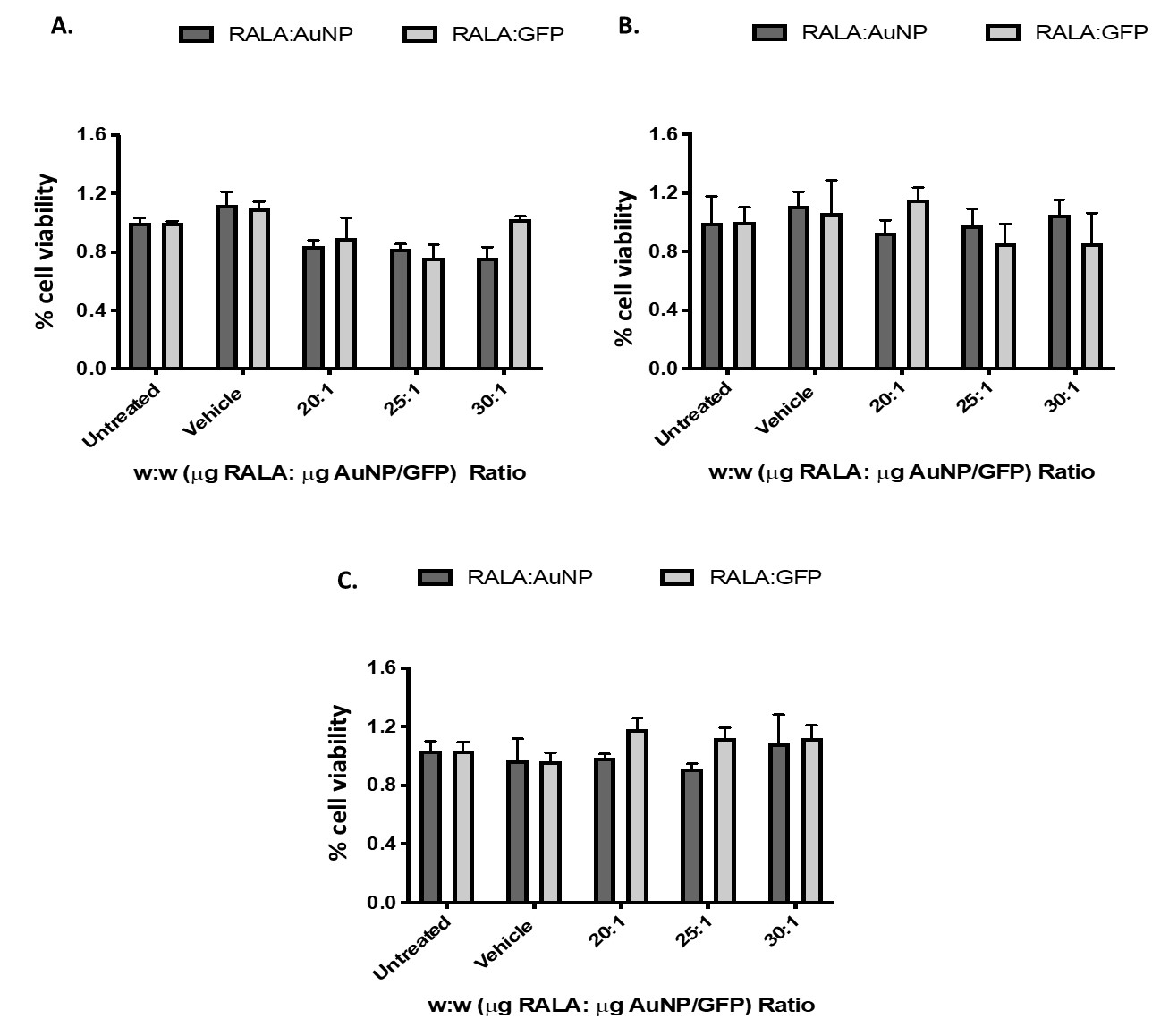
**

Figure S2

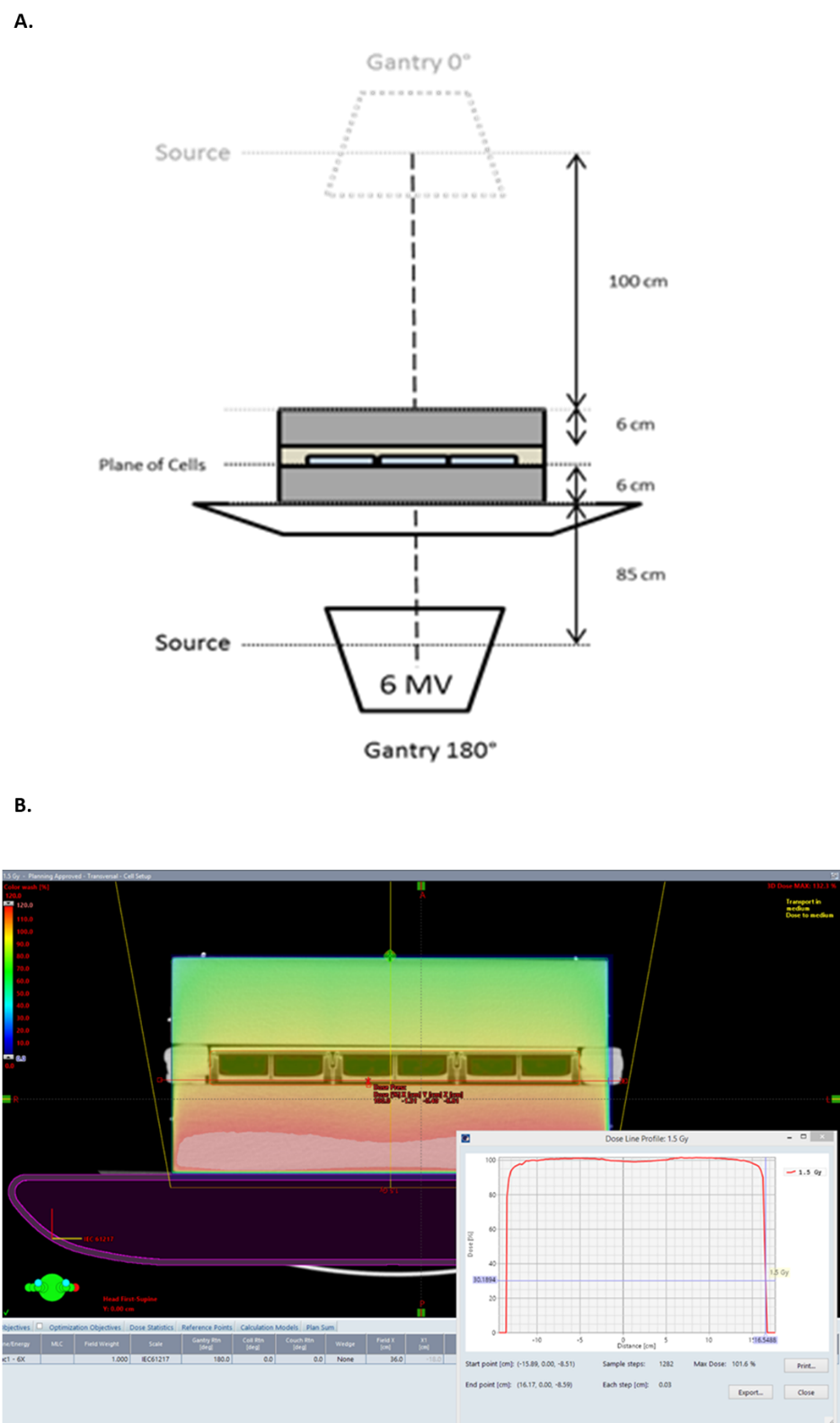
Figure S3

**Figure S4**

**
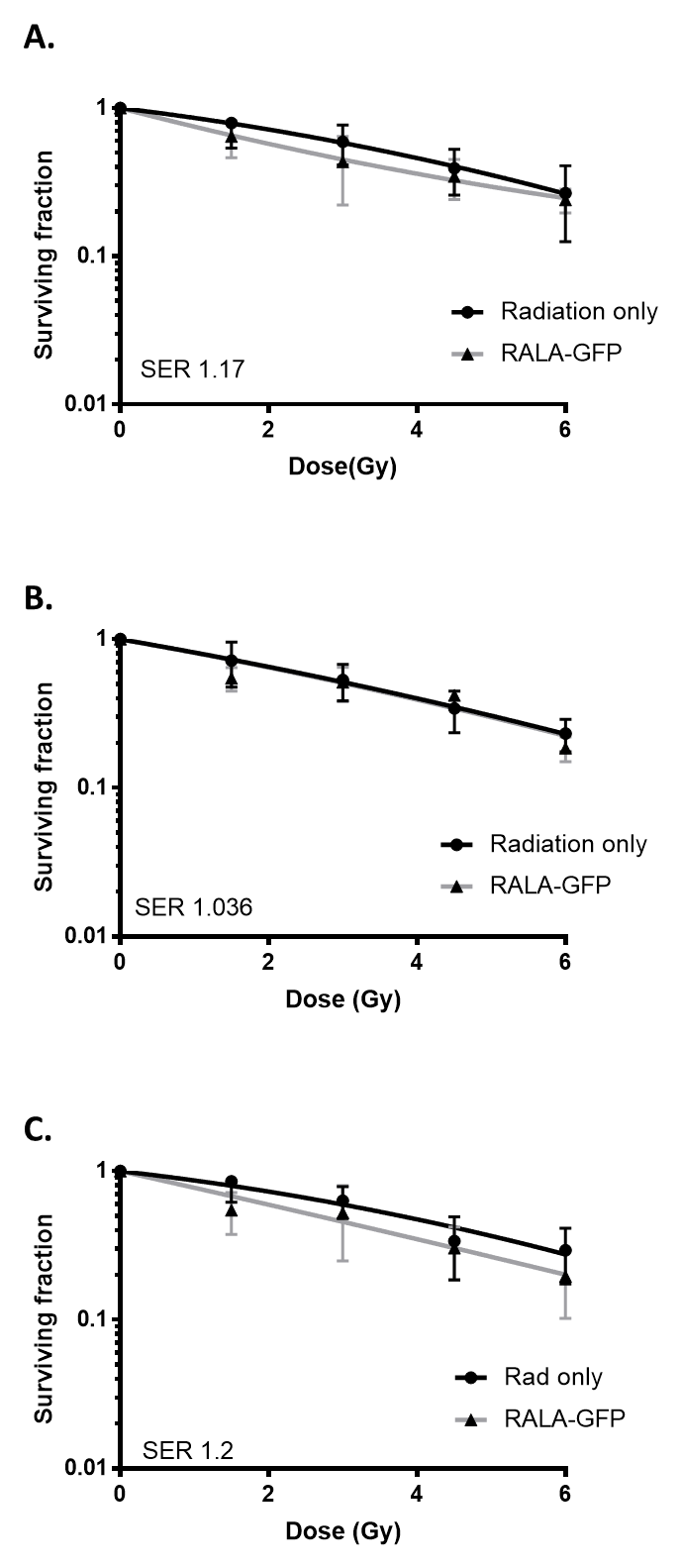
**

**Figure S5**

**
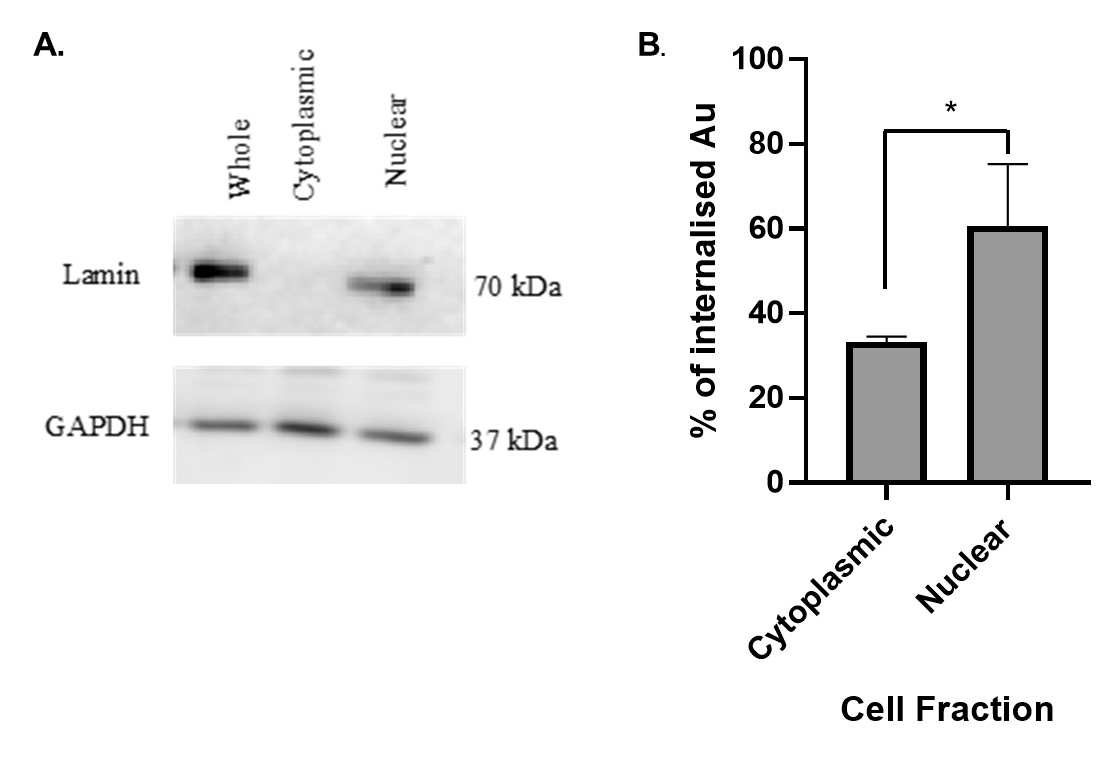
**

**Table S1.**

|  | **Z-Average (nm)** | **PDI** | **Zeta Potential (mV)** |
| --- | --- | --- | --- |
| **Citrate-AuNP** | 15.3 ± 0.8 | 0.3 ± 0.01 | -23.7 ± 3.8 |
| **5 µg RALA: 1µg AuNP** | 1868.8 ± 67.3 | 0.37 ± 0.02 | -3.6 ± 3.5 |
| **10 µg RALA: 1µg AuNP** | 3137± 567.9 | 0.34± 0.05 | 5.1± 8.1 |
| **15 µg RALA: 1µg AuNP** | 1653.7± 108.4 | 0.37± 0.01 | 10.6± 2.3 |
| **20 µg RALA: 1µg AuNP** | 107.7± 14.3 | 0.49± 0.05 | 16.7± 4.5 |
| **25 µg RALA: 1µg AuNP** | 107.9± 12.4 | 0.48± 0.06 | 17.8± 2.8 |
| **30 µg RALA: 1µg AuNP** | 103.1± 12.4 | 0.52± 0.03 | 21.5± 2.2 |

Table S2.

| Treatment Group | Doubling Time (Days) |
| --- | --- |
| Untreated | 3.3 +/- 0.64 |
| Citrate-AuNP (1 µg ) | 5 +/- 0.503 |
| RALA-AuNP (25 µg RALA: 1 µg ) | 4.2 +/- 0.79 |
| Radiation (8 Gy) | 8.3 +/- 3.8 |
| Citrate-AuNP + Radiation (8 Gy) | 10.02 +/- 2.52 |
| RALA-AuNP+ Radiation (8 Gy) | Not Reached |
